# Implications for the mammalian sialidases in the physiopathology of skeletal muscle

**DOI:** 10.1186/2044-5040-2-23

**Published:** 2012-11-01

**Authors:** Alessandro Fanzani, Alessandra Zanola, Fiorella Faggi, Nadia Papini, Bruno Venerando, Guido Tettamanti, Maurilio Sampaolesi, Eugenio Monti

**Affiliations:** 1 Department of Biomedical Sciences and Biotechnologies and Interuniversitary Institute of Myology (IIM), University of Brescia, Viale Europa 11, 25123, Brescia, Italy; 2 Department of Medical Biotechnology and Translational Medicine, University of Milan, Segrate, Milan, Italy; 3 Laboratory of Stem Cell for Tissue Engineering, IRCCS Policlinico San Donato, San Donato Milanese, Milan, Italy; 4 Stem Cell Research Institute, University Hospital Gasthuisberg, Herestraat 49, 3000, Leuven, Belgium; 5 Human Anatomy Section, University of Pavia, Via Forlanini 8, 27100, Pavia, Italy

**Keywords:** Sialidases, Gangliosides, Glycoproteins, Myogenesis, Skeletal muscle

## Abstract

The family of mammalian sialidases is composed of four distinct versatile enzymes that remove negatively charged terminal sialic acid residues from gangliosides and glycoproteins in different subcellular areas and organelles, including lysosomes, cytosol, plasma membrane and mitochondria. In this review we summarize the growing body of data describing the important role of sialidases in skeletal muscle, a complex apparatus involved in numerous key functions and whose functional integrity can be affected by various conditions, such as aging, chronic diseases, cancer and neuromuscular disorders. In addition to supporting the proper catabolism of glycoconjugates, sialidases can affect different signaling pathways by desialylation of many receptors and modulation of ganglioside content in cell membranes, thus actively participating in myoblast proliferation, differentiation and hypertrophy, insulin responsiveness and skeletal muscle architecture.

## Review

### The family of sialidases

Sialidases or neuraminidases (EC 3.2.1.18, systematic name: acetylneuraminyl hydrolase) are glycohydrolases widely distributed in nature, from viruses and microorganisms such as bacteria, protozoa and fungi, to vertebrates [1,2]. They catalyze the removal of the acidic sugar sialic acid from a great variety of gangliosides and glycoproteins, generally termed glycoconjugates, which are mainly exposed on the cell surface or are secreted in the extracellular matrix (ECM) [3]. The particular chemical structure, the terminal position on oligosaccharide antennas and the negative charge of sialic acid residues confer special structural properties to glycoconjugates, accounting for their biological relevance as key regulators of molecular and cellular interactions [4]. Depending on their ability to act on α-(2→3)-, α-(2→6)-, α-(2→8)-glycosidic linkages of terminal sialic acid residues, or on α-(2→8)-sialosyl linkages in oligo- and poly-sialic acids, sialidases are subdivided into exo- or endo-sialidases, respectively [2,4]. Since the first cloning of a mammalian sialidase in 1993 boosted the research on these enzymes [5], four distinct genes have been identified, encoding the lysosomal NEU1, the cytosolic NEU2, the plasma membrane-associated NEU3 and the mithocondrial/ER-associated NEU4 types, as summarized in various general reviews [2,6-8]. All of them behave as exo-sialidases and have a significant degree of homology, sharing with microbial sialidases typical amino acid motifs, such as the Y(F)RI(V)P motif in N-terminus and the so called Asp boxes (SxDxGxxΦ, where Φ stands for an aromatic residue) along the primary structure [2]. Over the past two decades, the involvement of NEU1, NEU2 and NEU3 in the physiopathology of skeletal muscle has clearly emerged from different studies, emphasizing that these enzymes may affect the life of muscle cells by modifying the cell content of sialylated lipids and proteins.

### The substrates of sialidases: gangliosides and glycoproteins

To understand the role of sialidases in skeletal muscle, we need to focus on their preferred substrates, which are gangliosides and glycoproteins. Gangliosides are acidic glycosphingolipids most abundant in the nervous system [9-11], but also present in skeletal muscle [12-16]. They are anchored on the outer leaflets of cell surfaces, with the sphingosine and fatty acid chains of the ceramide moiety embedded in the plasma membrane and the sugar oligosaccharide chain with terminal sialic acid(s) protruding toward the extracellular surface [17]. Gangliosides are involved in a plethora of physiological processes, such as cell-cell recognition and adhesion [18] and regulation of signal transduction in *caveolae*[19], lipid rafts [20] and glycosphingolipid-enriched microdomains [21]. They are also involved in the pathology of many diseases, such as the Guillain-Barre syndrome caused by an auto-immune response to surface gangliosides [22], influenza [23], some lysosomal storage diseases including Tay-Sachs disease (GM1 gangliosidosis), Sandhoff disease (GM2 gangliosidosis), sialidosis and galactosialidosis [24,25], an infantile-onset symptomatic epilepsy syndrome caused by ganglioside GM3 deficiency [26], type 2 diabetes [27] and Alzheimer’s disease [28,29]. Interestingly, altered GM3 levels have been recently found in the Hereditary Inclusion Body Myopathy (HIBM, also known as Distal Myopathy with Rimmed Vacuoles), a unique autosomal recessive muscle disorder characterized by adult-onset of muscle weakness in upper and lower limbs [30]. At the molecular level, gangliosides may participate in the composition and organization of membranes [31] and serve as modulators for several receptor proteins, usually limiting their activities. Ganglioside GM3, one of the essential components of plasma membrane rafts [32], is a negative regulator of insulin receptor (IR), as mice lacking GM3 display enhanced insulin sensitivity [33]. In addition, the coordinate enzymatic activity of sialidases and other glycohydrolases toward gangliosides may generate bioactive sphingolipids, such as ceramide, sphingosine and sphingosine-1-phosphate [34,35], which have a variety of important effects on the activation of muscle resident stem cells, regulation of contractile properties, insulin responsiveness and muscle fiber nourishment [36].

Beyond the gangliosides, sialidases recognize and desialylate glycoproteins involved in various functions, thus influencing many processes in different cell types [37]. Protein glycosylation may be of great importance during myogenesis, as confirmed by the fact that its inhibition impairs the fusion of myoblasts [38], but it is particularly important to preserve the integrity of musculoskeletal tissues in the post-natal age. In this regard, it is well accepted that the aberrant glycosylation on the mucin domain of α-dystroglycan, a member of the dystrophin-associated glycoprotein (DAG) complex [39,40], is associated with many forms of muscular dystrophy [40-43], commonly referred to as Dystroglycanopathies [44-46]. Recently, it has been shown that sialidase NEU1 deficiency causes muscle degeneration due to lack of processing toward yet unknown protein substrates [47], thus underlining the importance of sialidases in preserving the integrity of skeletal muscle by regulation of protein glycosylation.

Finally, free unbounded sialic acids are considered as regulators that modulate the function of several voltage-gated potassium and sodium channels responsible for the generation of action potentials in myofibers, cardiomyocytes and neurons [48,49]. On the basis of these preliminary observations, the ability of sialidases to modulate the cell contents of sialylated molecules have important consequences on muscle homeostasis, as described more in detail below.

### Functional role of sialidases in skeletal muscle

**Figure 1:**
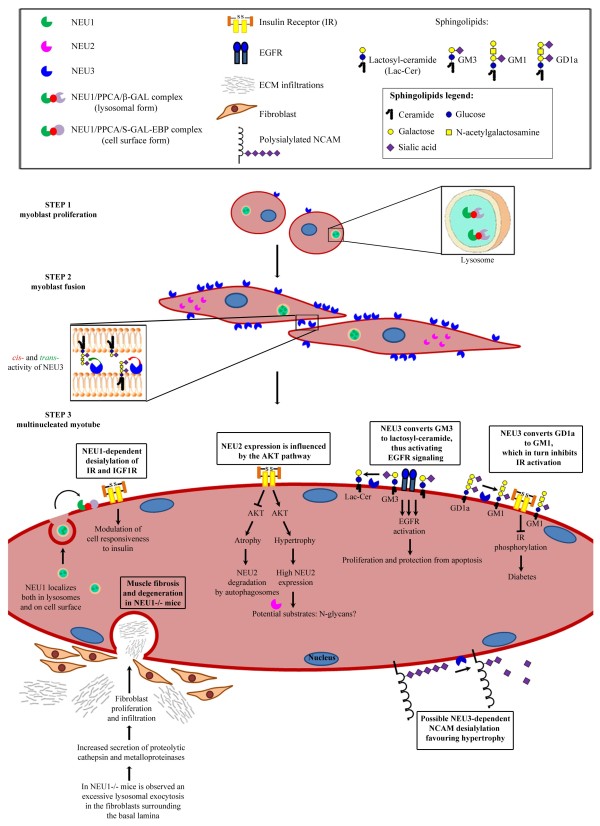
**Cartoon depicting the role of sialidases during the multistep process of myogenesis.** STEP 1: During myoblast proliferation, NEU1 is complexed with PPCA and β-GAL and its activity is mainly detectable within lysosomes, whereas plasma membrane-associated NEU3 is present at low levels. On the other hand, the cytosolic NEU2 is absent. STEP 2: Myoblast fusion is characterized by an early and transient increase of NEU1 expression as well as by a long-lasting increment of both NEU2 and NEU3 expression, the latter being involved in cell-cell recognition by working on gangliosides resident on the same cell (*cis*-activity) or on adjacent cells (*trans*-activity), as shown in the enlarged box. STEP 3: In differentiated myotubes, NEU1 participates in the degradation of sialo-glycoconjugates in lysosomes but it is also targeted to the cell surface, where it may desialylate IR and IGF1R, thus affecting their responsiveness to insulin. Since NEU1 limits the lysosomal exocytosis in the fibroblasts surrounding the myofibers, NEU1 −/− mice exhibit muscle degeneration due to infiltration of connective tissues. In the cytosol of myotubes, NEU2 expression is modulated mainly through the AKT pathway during hypertrophy and atrophy. In this compartment, cytosolic N-glycans may represent suitable substrates of this enzyme. At the plasma membrane, NEU3 positively affects EGFR signaling by converting GM3 to lactosyl-ceramide, while it blocks the IR activity by conversion of GD1a to GM1. Cell surface sialylated molecules, such as NCAM, may be a target of NEU3 activity. Note that depiction of sugar chains corresponds to the simplified style used in [144]

The following sections briefly summarize the main biochemical features and biological functions of NEU1, NEU2 and NEU3 and then discuss in detail their role in skeletal muscle (see also Table 1 and Figure 1), with the exception of NEU4 that, at present, seems to be less involved in muscle development and/or physiology.

**Table 1:** Sialidases in skeletal muscle

	**NEU1**	**NEU2**	**NEU3**	**NEU4**
Human chromosomal localization	6p21.31	2q37.1	11q13.5	2q37.3
Human disorders due to inherited deficiency	Sialidosis and Galactosialidosis [55]	none	none	none
Sialidase animal models	NEU1 −/− mice exhibit muscle degeneration [47]	none	Transgenic NEU3 mice develop insulin resistance [126]	none
Expression in myoblasts	*In vivo* and *in vitro*[47,83,84]	*In vitro*[5,89,92,93,97-99,103,104,107]	*In vivo* and *in vitro*[126,137]	not detected
Role proposed in muscle cells	NEU1 regulates the ECM deposition in skeletal muscle by limiting the lysosomal exocytosis in the fibroblasts sorrounding the myofibers [47]	NEU2 silencing prevents myoblast differentiation of rat L6 myoblasts [99]	NEU3 behaves as a negative regulator of glucose uptake [126]	
	NEU1 can desialylate both IR or IGF1R and influence insulin responsiveness [82]	NEU2 over-expression enhances C2C12 differentiation [98]	NEU3 is involved in C2C12 myoblast fusion by controlling the levels of GM3 [137]	
	NEU1 expression increases during the early stages of mouse C2C12 myoblast differentiation [83]	NEU2 expression increases through the PI3K/AKT pathway during differentiation and hypertrophy of C2C12 myotubes [103,104]	NEU3 over-expression delays differentiation but finally promotes the formation of hypertrophic myotubes [138]	
	NEU1 over-expression impairs C2C12 differentiation [84]	NEU2 is degraded through an autophagic-dependent pathway during atrophy of C2C12 myotubes [104,107]		
Muscle-derived tumors: rhabdomyosarcomas	-	NEU2 expression is undetectable in the human embryonal RD cells [109]		

### Lysosomal sialidase NEU1

NEU1 is a pivotal enzyme required to initiate the degradation of sialo-glycoconjugates in lysosomes [50], with preference for oligosaccharide and glycopeptide substrates [51], even if the protein can hydrolyze gangliosides in the presence of detergents or the sphingolipid activator Saposin B [52,53]. Newly synthesized NEU1 is transported to lysosomes, where is complexed with β-galactosidase (β-GAL) and the serine carboxypeptidase protective protein/cathepsin A (PPCA), the latter being specifically required for its catalytic activity [54]. Two genetically distinct lysosomal storage diseases are associated with NEU1 deficiency: sialidosis (OMIM 256550), which results from structural mutations at the NEU1 locus on chromosome 6p21 [55], and galactosialidosis (OMIM 256540), which is caused by a primary defect of PPCA, leading to secondary and combined deficiencies of NEU1 and β-GAL [56,57]. Depending on the levels of residual enzyme activity, NEU1 deficiency promotes a progressive lysosomal accumulation of sialylated glycopeptides, gangliosides and oligosaccharides in several cell types, leading to a broad spectrum of clinical manifestations such as abnormal somatic features, severe neurologic involvement and muscular dysfunction due to muscle hypotonia, atrophy, and osteoskeletal deformities [58]. These clinical symptoms have suggested an important role for NEU1 in muscle homeostasis, as deduced from the analysis of an inbred SM/J mouse strain with defects in NEU1 expression [59-61] and from the muscular phenotype of NEU1−/− mice [47]. Unlike dystrophic muscles, commonly characterized by sarcolemmal damage, intense inflammatory response, cellular necrosis and repeated cycles of muscle regeneration, the muscle pathology of NEU1−/− mice was derived from an excessive lysosomal exocytosis in the fibroblasts surrounding the basal lamina, leading to increased secretion of proteolytic lysosomal cathepsins and metalloproteinases that facilitate fibroblast proliferation and infiltration within the juxtaposed myofibers [47]. Lysosomal exocytosis, a calcium-dependent mechanism that allows the fusion of specialized lysosomes in the plasma membrane and the release of their luminal content extracellularly [62-65], is required during various physiological and pathological processes, such as the repair of the plasma membrane, the elimination of pathogenic bacteria or the release of HIV from infected cells [66-69]. In this context, NEU1 was previously found to negatively regulate the lysosomal exocytosis in hematopoietic cells by processing the lysosomal-associated membrane protein-1 (LAMP-1) protein [70]. In skeletal muscle NEU1 plays a critical role in the control of ECM deposition by limiting the lysosomal exocytosis in fibroblasts surrounding the myofibers, although the molecular targets underlying this mechanism remain to be elucidated. It is worth remembering that dystrophic muscles are usually characterized by the occurrence of fibrotic areas worsening the clinical outcome of patients. In this regard, it may be important from a therapeutic point of view to assess whether NEU1 may possibly influence by receptor desialylation certain signaling pathways playing a key role in muscle fibrosis, such as those dependent on the transforming growth factor beta (TGF-β) ligands [71-75]. Beyond the lysosomes, NEU1 can indeed be targeted to the cell surface in a multiprotein complex [76], playing an active role in the desialylation of several canonical and non-canonical cell receptors [77,78]. For instance, NEU1-mediated desialylation of integrin β4 leads to metastasis suppression in colon cancer [79,80], whereas in arterial smooth muscle cells the NEU1-dependent desialylation of both platelet-derived growth factor receptor (PDGFR) and insulin-like growth factor-1 receptor (IGF1R) lowered their intracellular signals, thereby diminishing cell proliferation [81]. In agreement with this evidence, the NEU1-mediated desialylation of both insulin receptor (IR) or IGF1R in L6 skeletal myoblasts leads to an increased or decreased cell proliferation in response to low or high concentrations of insulin, respectively, suggesting that NEU1 activity may influence glucose uptake in myoblasts [82].

As well as having a central role in the control of muscle architecture and responsiveness, NEU1 has been implicated in the regulation of myogenesis, as deduced from different *in vitro* studies. In this respect, it was shown that the NEU1 promoter region contains a CCAAT box and four E-boxes which serve to drive its expression through a MyoD-dependent mechanism [83]. In particular, NEU1 expression is closely and temporally up-regulated during the early stages of skeletal muscle differentiation, while it is down-regulated in the late stages through repression of the promoter activity by a MEK1-dependent mechanism, suggesting that NEU1 levels must be finely tuned [84]. In support of this gene regulation, constitutive NEU1 over-expression impaired the differentiating process of C2C12 myoblasts [84], suggesting that its constitutive over-activation may irreversibly affect the sialylation state and activity of some surface molecules involved in myogenesis, such as CD45, CD164 and the neural cell adhesion molecule (NCAM) [85-88].

Overall, we may conclude that NEU1 plays a pleiotropic role in skeletal muscle, encompassing the control of cell proliferation, differentiation and, above all, skeletal muscle architecture.

### Cytosolic sialidase NEU2

In 1993 Miyagi *et al.* reported the first molecular cloning of a mammalian sialidase encoding the sialidase NEU2 [5], a cytosolic enzyme expressed predominantly in skeletal muscle [89] and, to a lesser extent, in liver [90] and thymus [91]. Two subsequent works confirmed that the human NEU2 enzyme, sharing a high degree of homology with the rodent forms, is expressed in skeletal muscle and shows a subcellular localization and biochemical properties typical of the soluble sialidases [92,93]. Protein crystallography studies revealed that the human NEU2 consists of a six bladed β-propeller [94], a structural organization that is typically conserved between viral and microbial sialidases [95]. Furthermore, a detailed kinetic characterization has indicated that NEU2 exhibits a broad substrate specificity toward gangliosides and several different glycoproteins [96]. On the basis of sequence homologies, it is plausible to assume that the other mammalian sialidases share with NEU2 a conserved structural organization and a similar broad substrate specificity. Because of its almost exclusive expression in musculoskeletal tissues, two initial studies carried out in 1995 [97] and later in 2003 [98] pointed out the NEU2 involvement during differentiation of skeletal myoblasts *in vitro*. In this regard, an increased NEU2 gene transcription in rat L6 myoblasts appeared to be dependent on the presence in the promoter region of two pairs of E-box sequences, which are known binding sites for muscle-specific transcription factors involved in differentiation [97]. Subsequently, a similar transcriptional NEU2 up-regulation was observed during differentiation of mouse C2C12 myoblasts [98]. Indeed, a long-lasting increase of NEU2 enzymatic activity can be detected in the cytosolic fractions of myoblasts that are committed to differentiate [98,99]. These initial studies also discovered that altering NEU2 expression may influence the process of differentiation, as NEU2 suppression impaired the fusion process of L6 myoblasts [99], while over-expression of a rat form inhibited the proliferation and improved the differentiation in mouse C2C12 myoblasts [98]. To gain further insights, NEU2 has been studied during hypertrophy and atrophy. In this regard, the hypertrophy of C2C12 myotubes obtained via administration of IGF1 [100], vasopressin [101] or histone deacetylase inhibitor Trichostatin A [102] resulted in a significant increase in NEU2 transcriptional and enzymatic levels [103,104]. Moreover, such an increase is obtained through the activation of the PI3K/AKT signaling pathway, the master controller of the balance between protein synthesis and degradation in skeletal muscle [105,106]. In this regard, the increase of NEU2 activity assayed in hypertrophic C2C12 myotubes as a result of the constitutive AKT signaling was particularly impressive [103], suggesting that NEU2 may cooperate as one of its downstream effectors in building skeletal muscle. In agreement with this evidence, the atrophy of myotubes induced by nutrient deprivation or treatment with the glucocorticoid dexamethasone was relative to the impaired activity of the PI3K/AKT pathway and was characterized by a macroautophagic-dependent degradation of NEU2 [104,107]. In this context, *in vitro* assays showed that NEU2 is sensitive to the proteolytic action of the lysosomal cathepsins B and L [107], two isoforms with prevalent expression in skeletal muscle [108]. Finally, it is interesting to note that NEU2 was also investigated in human RD cells [109], which are cancerous cells deriving from rhabdomyosarcomas, a class of pediatric soft-tissue tumors composed of cell elements committed toward a myoblast lineage [110-113]. NEU2 was undetectable in RD cells and their partial myogenic differentiation obtained using drug treatments was not sufficient to restore its expression [109]; this suggests that the loss of NEU2, contributing to the impaired myoblast differentiation, may favor the oncogenic process. Overall, this growing body of evidence underlines an important role of NEU2, although not fully understood, in the fusion of myoblasts and the growth of myofibers. In support of this notion, it is intriguing that the impaired muscle regeneration observed in the SJL mouse, a model for human dysferlinopathy, is characterized by NEU2 down-regulation [114]. With regard to its potential substrates, it is believed that they should preferentially be glycoproteins instead of gangliosides, given the cytosolic localization of the enzyme. In this respect, it is of interest that NEU2 may participate in the degradation of complex-type N-glycans in the cytosol of MKN7 and MKN45 stomach cancer cells [115]. N-linked glycosylation is the most common type of post-translational modification playing a central role in the onset of some muscular dystrophies [43,44,116], as confirmed by the fact that mutations in DPAGT1, an essential enzyme catalyzing the first committed step of N-linked protein glycosylation, are responsible for a limb-girdle congenital myasthenic syndrome with tubular aggregates [117]. From this perspective, it is plausible that NEU2 may facilitate the process of myogenesis by ensuring the correct turn-over of glycosylated proteins through the processing of free oligosaccharides or misfolded glycoproteins that are released into the cytosol.

Altogether, these observations indicate that further investigation is required to understand the role of NEU2, also taking into account the fact that its expression in human muscles appears to be significantly lower in comparison to murine muscles.

### Plasma membrane-associated sialidase NEU3

NEU3, originally described as plasma membrane ganglioside sialidase [118-121], is a peripheral or extrinsic membrane-associated enzyme that has the ability to work on gangliosides that are located on the same membrane (*cis*-activity) or on the membrane of adjacent cells (*trans*-activity) [122,123], thus playing a central role in cell-cell interactions. As a result of NEU3 activity, the cell content of gangliosides decreases, leading to (i) a modification of the negative charge on glycocalix, (ii) a modulation of the cell content of bioactive lipids such as GM3, lactosyl-ceramide, glucosyl-ceramide and ceramide [10,34,35,124], (iii) a variation of the chemico/physical properties of lipid rafts whose clustering represents a pivotal step for membrane fusion during myogenesis [125] and (iv) a modulation of the activity of many canonical and non-canonical receptors, such as IR [126], epidermal growth factor receptor (EGFR) [127] and integrin β4 [128]. Regarding the latter point, NEU3 has been proposed as a cancer marker, because its up-regulation was found to promote the suppression of cell apoptosis in human tumors by a mechanism dependent on the depletion of gangliosides and subsequent over-activation of mitogenic receptors [127-130]. At the plasma membrane, NEU3 specifically localizes in lipid rafts and *caveolae*[131,132], microdomains specialized in the recruitment of molecules required for insulin signaling [133]. Two distinct works have indeed shown that NEU3 configures as an important regulator of this signaling along the skeletal muscle/liver axis. In particular, in skeletal muscle, NEU3 behaves as a negative regulator of glucose uptake, as in response to insulin the enzyme was activated by tyrosine phosphorylation and association with the growth factor receptor-bound protein 2 (GRB2), leading to accumulation of GM1 and GM2 gangliosides which, in turn, reduced the IR phosphorylation [126]. Accordingly, increasing both NEU3 expression or GM1 and GM2 levels in the plasma membrane of L6 myocytes and 3T3-L1 adipocytes inhibited IR phosphorylation [126]. Notably, mice over-expressing the human NEU3 enzyme developed a diabetic phenotype associated with hyperinsulinemia, islets hyperplasia and increased beta-cell mass [126]. Unlike what was observed in skeletal muscle, liver NEU3 over-expression positively improved insulin sensitivity and glucose tolerance in C57BL/6 and insulin-resistant KKAy mice by increased deposition of glycogen and triglycerides [134], suggesting that the effects of NEU3 on insulin responsiveness may differ between skeletal muscle and liver depending on the tissue-specific pattern of gangliosides.

Over the past years NEU3 has been characterized for its ability to enhance the activity of EGFR in two distinct ways, such as direct protein binding [127,130] or depletion of ganglioside GM3 [135,136]. In fact, NEU3 was found to promote myogenic differentiation of C2C12 myoblasts by specifically decreasing the amount of GM3, thereby allowing cell proliferation via EGFR signaling and protection from apoptotic stimuli [137]. In agreement with this evidence, NEU3 silencing led to an increased threshold of GM3 levels that caused EGFR inhibition, compromising the differentiation of C2C12 cells [137]. On the other hand, another recent study from the same group showed that NEU3 over-expression in C2C12 myoblasts resulted in a significant reduction of GM3 that initially slowed the differentiation by increasing cell proliferation, but then promoted the formation of hypertrophic myotubes [138]. This particular behavior could be explained by considering that the greater number of proliferating myoblasts derived from the enhanced EGFR signaling may be subsequently engaged in neoformed myotubes, which then display an increased number of myonuclei. In the same study [138], biochemical assays showed that NEU3 may cleave colominic acid, a linear α-(2→8)-linked polymer of sialic acid mirroring the negative antennas of NCAM, a molecule whose proper desialylation is crucial for myoblast fusion [88]. Based on these assumptions, it is intriguing to speculate that the hypertrophic behavior of NEU3-overexpressing myotubes could be related to the ability of NEU3 to desialylate specific surface molecules, such as NCAM.

Overall, NEU3 plays an important role in the control of insulin responsiveness, in addition to actively participating in the process of myogenesis by modulation of gangliosides and, presumably, other surface sialylated molecules involved in cell-cell recognition and fusion.

## Conclusions

Skeletal muscles are supplied from the fusion of multiple cell elements during embryonic development [139,140]. During the entire lifespan, their size mass is characterized by continuous remodelling as a result of physical exercise, chronic illnesses such as HIV, sepsis, diabetes and cancer cachexia, neuromuscular disorders or aging (sarcopenia) [141-143]. Sialidases have long been recognized as catabolic enzymes that, working within different subcellular compartments, can ensure the proper turn-over of glycoconjugates by catalyzing the removal of sialic acids residues. Nevertheless, a growing body of recent literature indicates that sialidases can specifically influence a number of different signaling pathways by modifying the cell content of gangliosides and sialylated receptorial and non-receptorial proteins. In skeletal muscle what we know is that the orchestrated expression of the lysosomal NEU1, cytosolic NEU2 and plasma-membrane NEU3 sialidases contributes to myoblast proliferation and differentiation, control of insulin responsiveness and regulation of tissue architecture by the correct assembly and deposition of ECM surrounding the myofibers, as schematically illustrated in Figure 1. However, there are many things we do not know about sialidases in various physiological and pathological conditions of skeletal muscle, and hopefully, cell and animal engineering tactics coupled with proteomics techniques will produce a more complete characterization of their substrates in order to fully understand their role.

In conclusion, we are confident that a more detailed understanding of the role of sialidases in skeletal muscle physiopathology may significantly contribute to open new exciting frontiers of basic and therapeutic exploration.

## Abbreviations

β-GAL: Beta-galactosidase; DAG: Dystrophin-associated glycoprotein; DPAGT1: Dolichyl-phosphate (UDP-N-acetylglucosamine) N-acetylglucosaminephosphotransferase 1; ECM: Extracellular matrix; EGFR: Epidermal growth factor receptor; GRB2: Growth factor receptor-bound protein 2; IGF1: Insulin-like growth factor-1; IGF1R: IGF1 receptor; IR: Insulin receptor; LAMP-1: Lysosomal-associated membrane protein-1; MEK1: MAPK/extracellular signal-regulated kinase kinase; NCAM: Neural cell adhesion molecule; PDGFR: Platelet-derived growth factor receptor; PI3K/AKT: Phosphoinositide-3-kinase/AKT; PPCA: Protective protein/cathepsin A; TGF-β: Transforming growth factor-beta.

## Competing interests

The authors declare that they have no competing interests.

## Authors’ contributions

AF and EM wrote the manuscript. AZ, FF, NP, BV, GT and MS edited the manuscript. All the authors read and approved the final manuscript.
